# Characterization of Polymeric Composites for Hydrogen Tank

**DOI:** 10.3390/polym15183716

**Published:** 2023-09-10

**Authors:** Waseem Gul, Yu En Xia, Pierre Gérard, Sung Kyu Ha

**Affiliations:** 1 Department of Mechanical Engineering, Hanyang University, 222 Wangsimni-ro, Seongdong-gu, Seoul 04763, Republic of Korea; mech487@hanyang.ac.kr (W.G.); xiayuen@hanyang.ac.kr (Y.E.X.); 2 Arkema, G.R.L., 64170 Lacq, France; pierre.gerard@arkema.com

**Keywords:** filament winding, hoop strength, hydrogen tank, stress concentration, ASTM D2290, thermoplastic resin, Elium^®^ 591, carbon neutrality, polymer composite

## Abstract

Carbon neutrality has led to a surge in the popularity of hydrogen tanks in recent years. However, designing high-performance tanks necessitates the precise determination of input material properties. Unfortunately, conventional characterization methods often underestimate these material properties. To address this limitation, the current research introduces alternative designs of ring tensile specimens, which enable accurate and reliable characterization of filament-wound structures. The advantages and disadvantages of these alternative designs are thoroughly discussed, considering both numerical simulations and experimental investigations. Moreover, the proposed ring tensile methods are applied to characterize thermoplastic composites for hydrogen storage tanks. The results indicate that the mechanical strengths and stiffness of carbon fiber-reinforced thermoplastic Elium^®^ 591 composites closely match those of epoxy-based composites. This newfound accuracy in measurement is expected to contribute significantly to the development of recyclable hydrogen tanks.

## 1. Introduction

Using fossil fuels as the dominant energy source for transportation has significantly impacted the Earth’s climate, posing a threat to life on our planet. Consequently, there is a global search for an alternative, environmentally friendly energy source to ensure humanity’s survival. Hydrogen has emerged as a promising energy carrier that is both ecologically sustainable and easily producible. Furthermore, hydrogen exhibits greater efficiency compared to conventional fuels, making it a highly hopeful solution for a wide range of energy needs.

The hydrogen economy has gained global recognition as a means of transitioning towards cleaner energy and addressing environmental issues. Different regions and countries are making ambitious strides in investing in hydrogen research, development, and deployment. Europe has emerged as a frontrunner, with Germany, the Netherlands, and Norway leading the way through comprehensive strategies and substantial investments. In Asia, Japan and South Korea have taken the lead in hydrogen technology advancement, focusing on production, utilization, and large-scale infrastructure projects. Australia, China, and the United States are also actively pursuing the development of their hydrogen economies. This dynamic progress on a global scale signifies a pivotal step towards a more sustainable and eco-friendly future.

However, the commercialization of hydrogen for fuel cell vehicles faces certain challenges. Ensuring the safe design of hydrogen storage tanks and their recyclability at the end of their useful life are among the obstacles to overcome. The safe design of storage tanks is crucial due to the nature of hydrogen’s flammability, and efforts are underway to develop secure storage solutions. Additionally, establishing efficient recycling methods for these tanks is essential to recover valuable materials and promote a circular economy approach. By addressing these challenges, the commercialization of hydrogen for fuel cell vehicles can be realized, contributing to a safer, more sustainable transportation sector.

**Figure 1:**
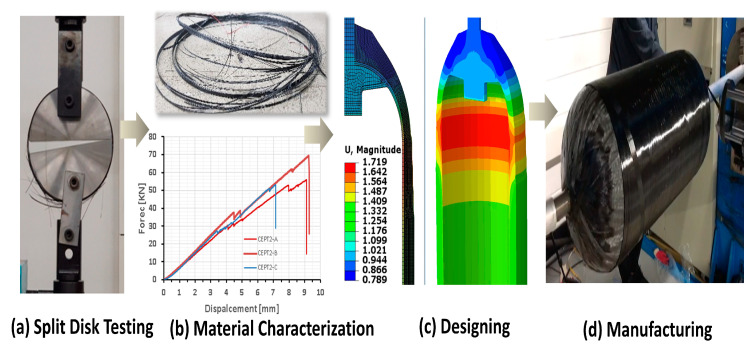
Research and Development flow chart of the hydrogen tank.

The carbon/resin properties in the UD directions are the key design factors to ensure a safe and economical hydrogen tank. Characterizing the composite material is an initial and crucial step in the research and development of the hydrogen tank, as shown in Figure 1. The accuracy of the material properties is vital to predicting tank behavior and burst pressure accurately. By convention, composite material properties are measured using the ASTM D2290 [1] split disk method, which uses hoop-wrapped rings loaded to failure in tension by split disks.

**Figure 2:**
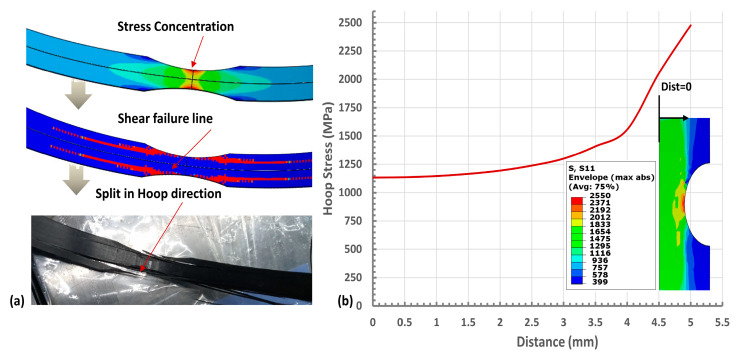
(**a**) Failure mode of the notched specimen (**b**) Non-uniform Stress distribution in the gauge area of the notched specimen.

However, hoop tensile strength measured through the notched specimen is always lower than the actual strength. Because the stress distribution in the notched specimen is not uniform, and there is a stress concentration in the notched region. This stress concentration causes in-plane shear stress, as shown in Figure 2, which results in splitting the specimen in the hoop direction much earlier than the fiber breakage. Therefore, it is impossible to accurately measure the fiber properties of the hoop-winded specimen using the existing test specimen design. Kaynak C. et al. [2] also observed that notched specimens with hoop winding tend to split along the hoop direction. In this case, debonding initiates at the notch, resulting in shear failure before fiber failure. Such failures tend to predict a lower tensile strength.

Another issue is that the failure along the ring’s circumference is forced to occur parallel to the disk split line during the test. The stress state at this site is not exclusively tensile under the given loading conditions. Bending stress is also generated during tensile testing, which produces non-uniform strain distribution along the specimen’s thickness direction, resulting in an apparent tensile strength rather than a true tensile. Considering the local bending in the split disk test, Zhao, Y et al. [3] recommended measuring the strain, which is used to calculate stiffness, in the area 10° away from the disk split, where local bending has no influence. Similar phenomena were observed by Lee et al. [4] during the testing of C/SiC specimens. They determined that the strength of the tubular specimen was about 26% lower than the plate specimen. Stress concentration and local bending in the gauge area are significant issues in notched ring design, resulting in lower tensile strength prediction. Abeda et al. [5] obtained hoop tensile strength and modulus of filament-wound carbon fiber-reinforced plastic (CFRP) composite rings using the split-disk test method. Results showed that the tensile strength and modulus of the CFRP pre-preg tow were about 20% lower than reported data. Ramesh et al. [6] conducted an experimental work for ultimate strain and tensile load carrying capacity of carbon fiber-reinforced composite (CFRC) and GFRC using a modified split disk test, and results were compared with uniaxial coupon tests and compression tests. Results presented that the ultimate strain and tensile strength were lower than the flat coupon test.

Likewise, Charan. et al. [7] experimentally demonstrated that the apparent tensile strength of a ring test is 10% to 30% lower than flat coupons’ results. Several other researchers [8,9] have investigated the split-disk test as a primary experimental method to evaluate circumferential mechanical properties of the filament wound components. However, some researchers also used non-standard tests and specimens. Such as Laiarinandrasana et al. [10] studied the stress concentration in the notched and complete ring specimen (without notched). Similarly, some researchers [11,12,13] used non-standard methods such as the burst ring and expanded-plug ring tests.

Another critical issue facing the hydrogen tank industry is the recyclability of hydrogen tanks at the end of their useful lives. Hydrogen tanks are traditionally manufactured using thermosetting resin, which requires a long curing cycle and is not environmentally friendly since it is not recyclable. Using thermoplastic resin in manufacturing hydrogen tanks can solve the issue of recyclability. However, the material properties of the thermoplastic-based composite are not readily available as in the case of the Epoxy-based composite. Although, some researchers [14] have determined the impact behavior of the thermoplastic-based composite for wind turbine blade application. However, no research has been conduct like this or keep it aed on determining the thermoplastic composite’s tensile strength and tensile modulus, which are crucial factors in designing the hydrogen tank.

In the last few years, improvements in winding methodologies and testing devices have been proposed to compute accurate mechanical properties for composite pressure vessels. However, there has been no consideration of redesigning ring tensile specimens. The design of the ring tensile specimen may be the main reason for the 20–30% lower strength prediction compared to the actual strength of the material due to stress concentrations and non-uniform stress distributions in the gauge section.

In this investigation, we conducted a comprehensive analysis of different designs for ring tensile specimens, thoroughly examining their respective advantages and limitations. Conventionally, the ring specimen design includes a notch to direct the failure to the gauge section. However, in the Unidirectional (UD) wound specimen, the notch leads to stress concentration, culminating in the specimen splitting along the hoop direction ahead of fiber breakage, as demonstrated in Figure 2. This premature splitting results in a tensile strength significantly lower than what is theoretically predicted.

To overcome these challenges, our updated designs omit the notch at the gauge section and incorporate tabs to maintain stress uniformity and direct failure toward the gauge section. In addition, we made a crucial design modification by transforming the curved gauge area into a flat one. Our numerical simulations indicate that these changes facilitate a more evenly distributed hoop stress throughout the gauge cross-section, leading to enhanced tensile strength. This is further corroborated by our experimental data, showing that the newly designed ring tensile specimens can withstand greater loads more consistently compared to their traditional notched counterparts. Furthermore, we utilized these novel ring tensile specimens to characterize filament-wound composites derived from Elium^®^ 591 resin [15], a thermoplastic resin that remains in a liquid state at room temperature.

## 2. Designing of Ring Tensile Specimen

### 2.1. Configuration of the Tensile Specimens

**Figure 3:**
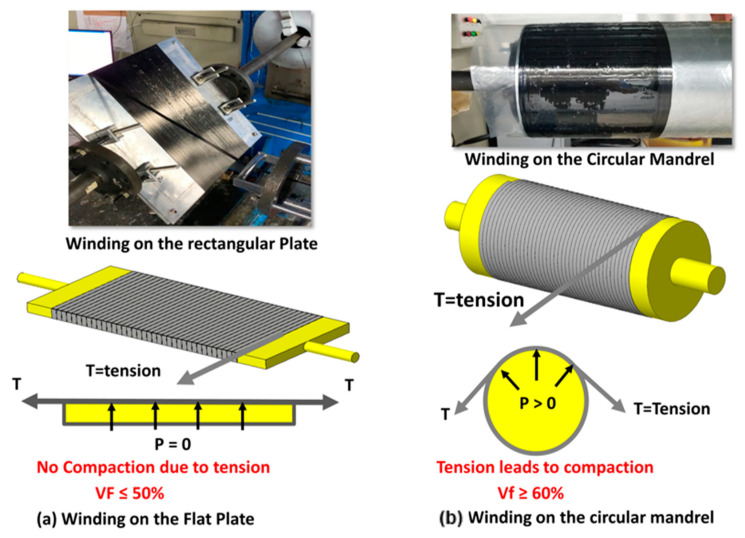
Winding of the tensile specimen (**a**) winding on the flat plate (**b**) winding on the circular mandrel.

Hydrogen tanks are manufactured with a filament winding process. To better characterize the tank structure, tensile specimens should be manufactured using the same process. A tensile specimen in the flat shape recommended by ASTM D3039 [16] and a ring shape recommended by ASTM D2290 can be manufactured using the filament winding process, as depicted in Figure 3. During the filament wing process, tension is applied to the winding rovings for better compaction and consolidation of the fiber. However, winding on the plate mandrel, its effect is insignificant because the applied tension’s normal vector is almost equivalent to zero, as shown in Figure 3a. On the other hand, tension applied on the circular mandrel leads to compaction of the fiber resulting higher volume fraction. A ring specimen manufactured with a filament winding process is preferred over a flat specimen due to the higher volume fraction.

**Figure 4:**
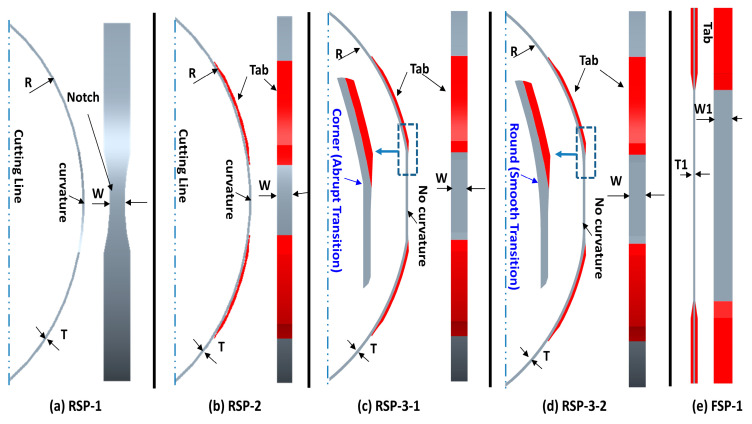
Design comparison of tensile specimen for characterization of the filament wound structure.

This research mainly focuses on designing an efficient ring tensile specimen. An efficient design of a ring tensile specimen can transfer 100% applied load to its gauge area. It also exhibits uniform stress distribution and failure in the gauge section. In the current research, several designs of ring tensile specimens are proposed, and their design effectiveness is evaluated numerically. Further design validations are made experimentally using split disk tensile testing as recommended by the ASTM D2290. The designs of the tensile specimen considered in this study are shown in Figure 4. Designs that appeared in Figure 4a–d are the ring types, and the design in Figure 4e is the flat-type tensile specimen. All ring-type specimens’ internal radius (R = 107.9) and gauge cross-sectional area (2 × T × W = 2 × 1.5 × 10) are kept constant in the numerical simulation for comparison purposes.

The RSP-1 is the conventional notched specimen widely used to characterize plastic or reinforced plastic pipe by the split disk method, as illustrated in Figure 4a. This specimen is manufactured using the filament winding process on a round mandrel, which gives a volume fraction of more than 60%. The notch in the RSP-1 specimen design is designed to concentrate the failure at the gauge section. This type of configuration helps predict the material properties of the isometric material and biaxially reinforced pipe and structure accurately. However, the notch in a unidirectional (UD) wound tensile specimen will cause severe damage along the fiber lines, as shown in Figure 2a, leading to an inaccurate measurement of hoop tensile strength. In this study, a notch of 85 mm radius is provided.

The RSP-2 specimen is newly proposed. It is also manufactured with a filament winding process. Unlike the RSP-1 design, there is no notch on the gauge area, as shown in Figure 4b. Instead, tabs are provided to obtain the uniform stress distribution and to concentrate the failure at the gauge section.RSP-2 design demonstrates the uniform stress distribution along the width direction compared to the RSP-1 specimen. However, the stress state at the gauge section is not purely tensile because of the bending moment imposed during the test at the split between the split disk test fixture. Due to the local bending, stress distribution at the gauge section along the thickness direction is not uniform, which may result in a lower prediction of the tensile strength.

The RSP-3-1 and RSP-3-2 designs are also newly developed. Unlike the RSP-1 and RSP-2 designs, the gauge section of these designs is flat instead of curved, as illustrated in Figure 4c,d. The purpose of the flat gauge section is to prevent the local bending moment enforced in the specimen due to curvature at the test fixture split during the tensile testing. With the absence of the bending moment stress state at the gauge section is purely tensile. Furthermore, tabs are also provided to smooth tensile load transfer to the gauge section and avoid stress concentrations in the transition area. This results in the prediction of true tensile strength.

**Figure 5:**
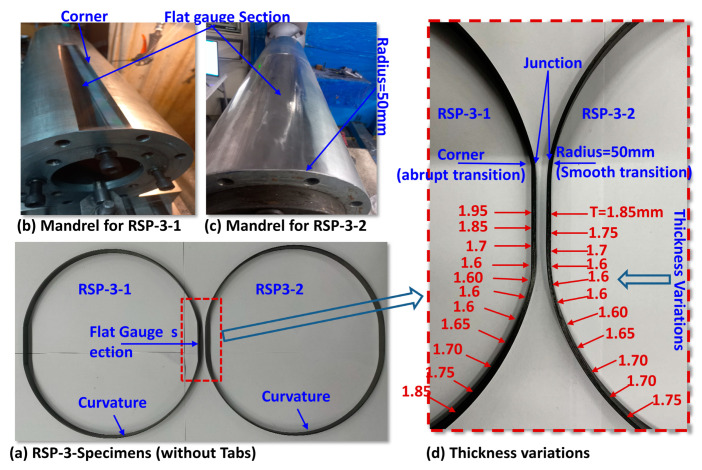
(**a**) RSP-3 specimen without tabs (**b**) Mandrel for RSP-3-1 (**c**) Mandrel for RSP-3-2 (**d**) Thickness Variations in the gauge section.

However, manufacturing the RSP-3 specimen is a complicated process compared to the other types.RSP-3 categories specimens are filament wound on the specially machined mandrel. The mandrels are machined to give about 40 mm flat length of the gauge section, as shown in Figure 5b,c. Two significant problems are associated with manufacturing: forming a sharp corner at the junction (transition between the flat gauge and curved region) and the non-uniform thickness at the gauge section, as shown in Figure 5d. The former issue is resolved by applying the 50 mm radius on the winding mandrel in Figure 5c. The latter can be controlled using the external compressive forces during the winding process on the flat gauge section as elaborated in the manufacturing of the specimen section. The specimens with sharp corners and smooth radii at the junctions are designated as RSP-3-1, and RSP-3-2, respectively, as shown in Figure 5a. RSP-3-2 is the advanced version of the RSP-3-1.

FSP-1 is a flat-type tensile specimen specified by ASTM D3039 [16] for determining polymer matrix composite materials’ tensile properties. It can also be manufactured using the plate mandrel with a filament winding process, as shown in Figure 3a. A flat-type specimen’s main disadvantage is its smaller volume fraction than a ring-type specimen. A comprehensive comparison of all specimen designs is made in Table 1.

### 2.2. Finite Element Analysis

**Figure 6:**
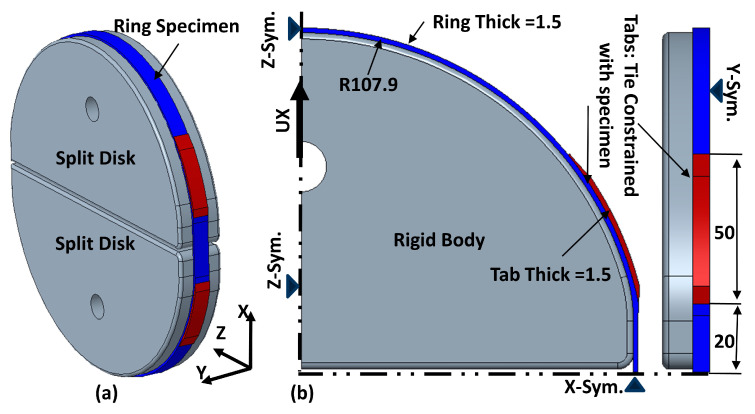
(**a**) Geometric model (**b**) Finite element model of ring tensile specimen.

Finite element analyses are performed to assess the effectiveness of the proposed designs of ring tensile specimens. The geometric assembly model of the split disk with ring specimen and corresponding one-eighth symmetric finite element models are shown in Figure 6a,b.

In the finite element model, the specimen and tab are modeled with five UD layers with an angle of 89.60° to simulate the actual hoop-winded specimen behavior. Each layer is assigned with UD lamina material properties of carbon-epoxy composite [17,18,19]. The material properties include Young’s modulus (E_1_ = 135 GPa, E_2_ = E_3_ = 9.66 GPa), Shear modulus (G_12_ = G_13_ = 5.86 GPa, G_23_ = 3.46 GPa), and Poisson’s ratio (ν_12_ = ν_13_ = 0.25, ν_23_ = 0.41). Both tabs and specimens are assigned identical material properties to avoid stress concentration due to stiffness differences

The specimen and tabs are meshed using SC8R continuum shell elements. That is a general-purpose element and suitable for nonlinear geometric analysis. The mesh density is fine-tuned in the gauge section and areas where stress concentrations are expected, like the end of the tab and the transition zone. The split disk is meshed using a general-purpose solid element C3D8R, and rigid body constraints are applied to them as there is no requirement to evaluate the stress and strain in the disk elements.

The symmetry boundary conditions are applied to the X, Y, and Z faces. A displacement boundary condition is applied along the Y direction on the reference point of the upper disk to simulate tensile loading, as shown in Figure 6b. A general contact algorithm simulates the contact behavior between the split disk and the tensile specimen. A coefficient of friction of 0.3 [20,21,22,23] is considered between the specimen and disk surfaces.

**Figure 7:**
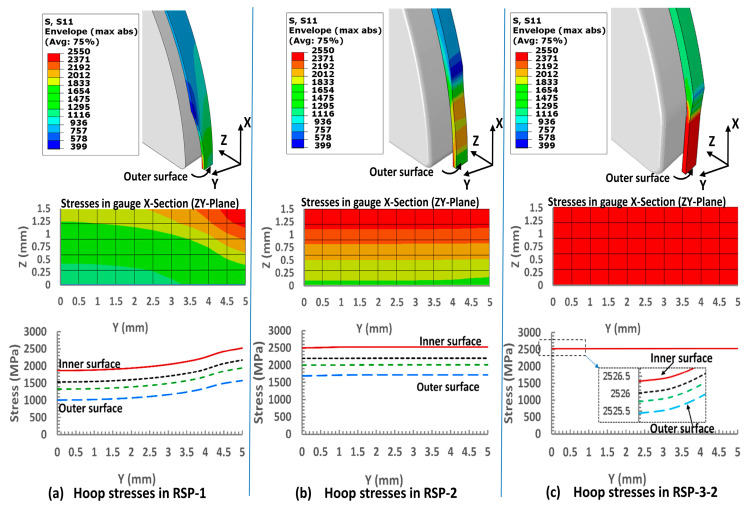
Hoop tensile Stress distribution in the gauge section of conventional notched design (RSP-1) and proposed designs (RSP-2 and RSP-3).

The results of the finite element analyses are illustrated in Figure 7. Please note that only ring tensile specimen results are shown as the intention of this research to design the ring specimen because it gives a higher volume fraction compared to the flat specimen, as shown in Figure 3. Furthermore, in the RSP-3 category, hoop stress distribution in the RSP-3-1 and RSP-3-2 are identical, and RSP-3-2 is the advanced version of the RSP-3-1. Only hoop stress distribution in RSP-3-2 is shown for comparison with other ring specimen designs.

Hoop tensile stress distributions are visually depicted by plotting stress profiles in the YZ plane at a cross-sectional area in the middle of the gauge section. Moreover, line graphs are employed to enhance the visualization of stress variations. These line graphs are generated based on four distinct nodal paths, extending from the outer surface to the innermost surface along the width of the gauge section. This approach provides a detailed representation of how the hoop tensile stresses evolve and vary within different regions of the gauge section. By examining these stress profiles and line graphs, researchers can gain insights into the distribution of hoop tensile stresses and make informed comparisons between different ring specimen designs.

As shown in Figure 7a, stress distributions in the notched Specimen (RSP-1) are non-uniform along the Y (width direction) and Z (thickness direction) directions. The values of the hoop tensile stresses are reduced gradually from the inner surface to the outer surface. The maximum stress of 2526 MPa is at the notch center on the innermost surface, and the minimum stress of 1009 MPa is at the center of the gauge section on the outer surface of the specimen. With the overall average stress in the cross-section being 1632 MPa, the stress concentration factor k from Equation (1) can be calculated as 1.54.

(1) $$K=\frac{\sigma_{max}}{\sigma_{nominal}}$$

where *σ_max_* is the maximum nodal hoop stress, and *σ_nominal_* is the average hoop stress on nodes in the gauge x-section. The high-stress concentration in the notched hoop specimen produces in-plane shear strain, which results in the splitting of the specimen in the hoop direction much earlier than fiber breakage, as shown in Figure 2a. In this case, the specimen shows significantly lower tensile strength.

Contrary to the notched specimen, the hoop stress distribution in the RSP-2 specimen is uniform along the Y (width direction), as shown in Figure 7b. However, the stress distribution is not uniform along the Z (thickness direction) direction. This nonuniformity of the stresses is due to the bending moment produced in the gauge section in the slit area of the split disk. The maximum hoop stress of 2509 MPa appeared on the innermost surface, and the minimum constant stress of 1683 MPa was on the outer surface. The average nodal stress in the gauge cross-section is 2112 MPa, 29% higher than the notched specimen (RSP-1). Such statistics make the RSP-2 specimen a suitable candidate for measuring the hoop tensile strength of the filament wound specimen. The tensile strength measured will also be called the apparent hoop tensile strength rather than the actual strength due to the involvement of the bending moment.

The RSP-3-2 specimen, distinguished by its flat gauge section, exhibits a uniform hoop tensile stress distribution, as shown in Figure 7c. Stress is distributed evenly along the Y and Z directions, contrasting with other ring designs, such as the RSP-1 specimen, which suffers from stress concentration and bending moment effects. The unique feature of a 40 mm flat portion in the RSP-3-2 specimen helps eliminate bending moment effects, resulting in a uniform stress distribution.

In addition, the specimen’s design includes tabs that ensure an even distribution of all applied forces across the gauge area, preventing stress concentration in the transition region. The gauge cross-section displays an average nodal stress of 2529 MPa, 54% higher than the notched specimen. This demonstrates effective force transfer to the gauge section, proving the RSP-3-2 design is an efficient design for testing the hoop tensile strength of filament-wound structures such as hydrogen tanks.

### 2.3. Tabs Effect

**Figure 8:**
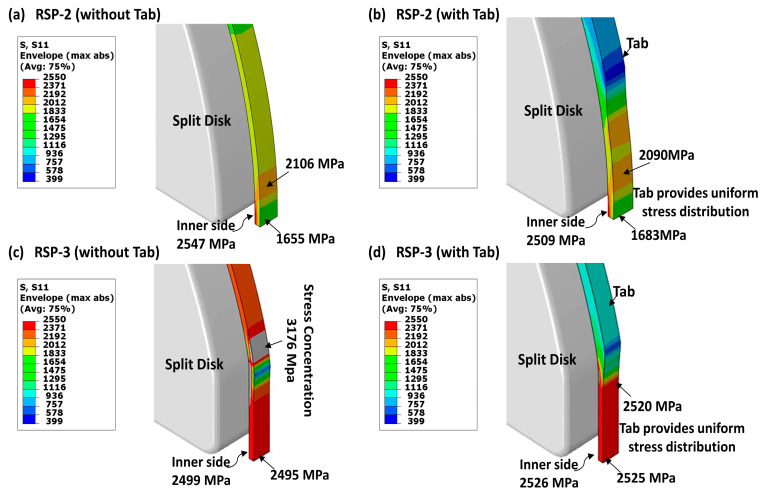
Effect of Tabs; (**a**,**b**) RSP-2 Design; (**c**,**d**) RSP-3 Design.

The tabs have been used on the ring tensile specimen for the first time to achieve uniform stress distribution and to concentrate failure in the gauge section only. Traditionally, composite materials are tabbed before testing for two reasons: to protect them from damage during the test and to increase the loading region, thereby reducing local stress concentrations. However, no researcher has studied the effect of tab in the ring tensile specimen. In the conventional design (RSP-1), a notch is provided to concentrate the failure at the gauge section. However, the Notch in the UD wound tensile specimen will cause severe damage along the fiber lines, as shown in Figure 2a, leading to an inaccurate measurement of UD tensile strength. The notch is replaced with tabs in the newly proposed designs (RSP-2, RSP-3-1, and RSP3-2). The purpose of the tabs is to eliminate stress concentration and provide uniform stress distribution at the gauge section, as illustrated in Figure 8.

Finite element analyses are employed to identify the most effective shape and placement of tabs on the specimen. In the RSP-2 specimen, the tabs are designed to ensure a smooth distribution of stresses along the thickness direction while directing failure specifically to the gauge section, as depicted in Figure 8b. On the other hand, the tabs in the RSP-3 design aim to eliminate the concentration of high stresses at the junction area, as shown in Figure 8c. Furthermore, these tabs facilitate the smooth transfer of stresses to the gauge area, ultimately enhancing the specimen’s load-carrying capacity.

## 3. Manufacturing of the Test Specimen

### 3.1. Liquid Thermoplastic Resin-Elium^®^ 591

Epoxy resin is the most used material in the high-pressure composite tank market. It provides good mechanical resistance to contain the high-pressure hydrogen inside the tank. However, like all thermoset composites, Epoxy has drawbacks, the most significant of which is its non-recyclability. Currently, end-of-life epoxy resin tanks, which cannot be recycled, are buried or recovered as solid fuel, especially for heating cement mills [15,24]. Due to tighter regulations regulating the burial of industrial waste and increased recycling obligations, thermoplastic resin Elium^®^ 591 by Arkema, France [25], is an alternate option for manufacturing hydrogen tanks.

Elium^®^ 591 resin consists of an acrylic base polymer diluted in a reactive monomer blend with processing additives so that it is very fluid at room temperature. The resin is mixed with a polymerization initiator to create a thermoplastic matrix with high molecular weight. Ommirad819 (a photoinitiator) and BCHPC (a thermal Peroxide) are mixed with resin with a specific ratio to initiate the curing process. Elium^®^ 591 is compatible with traditional manufacturing methods, and the parameters can be adjusted according to the requirements (viscosity, time, and temperature reactivity). Due to low viscosity, it is suitable for filament winding of hydrogen tanks, and its characteristics are equivalent to traditional Epoxy. Additionally, the Elium^®^ 591 resin tank can be recycled at the end of the tank’s life, and precious carbon fibers and the resin can be recovered for other uses.

### 3.2. The Filament Winding with Liquid Thermoplastic Resin

As discussed in the previous section, using thermoplastic resin can solve the problem of recyclability of the hydrogen tank and the global carbon neutrality issue. However, no research data are available on the mechanical characterization of Elium^®^ 591-based composite, which is necessary for designing the hydrogen tank.

**Figure 9:**
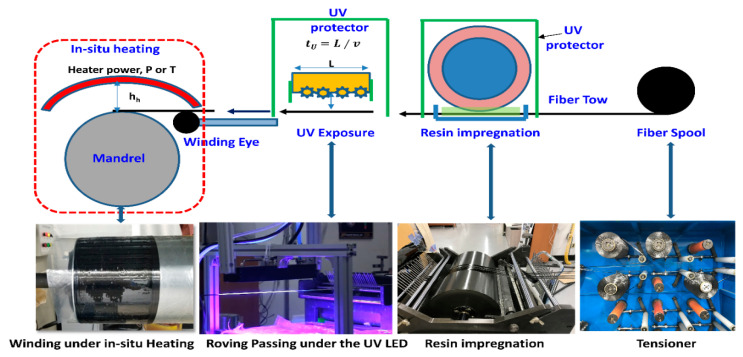
Filament winding process with thermoplastic resin (Elium^®^ 591).

Current research aims to characterize the Elium^®^ 591 base ring tensile specimen manufactured from the filament winding. The modifications are made in the existing epoxy-based filament winding process line to develop the ring tensile specimen with carbon fiber impregnated with Elium^®^ 591, as shown in Figure 9. The combined ultraviolet (UV) and in-situ thermal curing systems are accommodated in the production line. Combined Ultraviolet (UV) and in-situ thermal curing schemes are used for fast-curing purposes.

The water-cooled UV lamp of the LG 3535 series with a wavelength of 395 nm is retrofitted right after the resin bath. A safety shield is also integrated around the UV lamp system to ensure worker safety and proper operation of the winding machine. The UV lamp’s position in the production line, distance from the underneath moving fiber tows, and radiant power are calibrated to produce B-stage curing of the resin, as shown in Figure 9. This type of curing does not make full-cure resin. Still, it only gives tackiness to the resin, improving the consistency of the fiber-resin ratio and producing a complex winding pattern with a high winding angle. It also reduces the resin dripping from the mandrel and ensures a clean winding process. After this, B-stage curing fibers roving is wrapped on the rotating mandrel. The In-Situ thermal heating system is also provided to maintain a constant curing temperature of 85 °C over the rotating mandrel. 

To evaluate the potential of the Elium-based composite as a suitable alternative to epoxy composites for the fabrication of hydrogen tanks. We have created ring tensile specimens using both Elium and epoxy resin systems. It’s noteworthy that we maintained uniform winding conditions and specimen designs for both material groups to facilitate a valid and rigorous comparison.

The hoop ring tensile specimens of the thermoplastic composite are manufactured with Hyosung carbon fiber H2550-24K [26] and Elium^®^ 591resin system using a wet filament-winding process described in the previous section. Elium^®^ 591 resin is mixed with Ommirad 819 (a photoinitiator) and BCHPC (a thermal peroxide) to initiate the curing process. To manufacture thermoset composite specimens, epoxy resin systems with SE-700A-Epoxy and SH-150A-hardener are used to impregnate the H2550-24K carbon fiber during the conventional filament winding process line.

**Figure 10:**
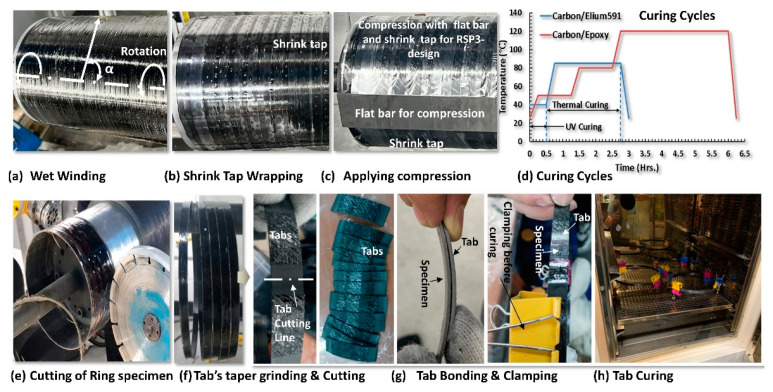
Ring Tensile Specimen Manufacturing Process.

The complete manufacturing process of the ring tensile specimens is illustrated in Figure 10. The filament winding was conducted on a cylindrical mandrel of 215 mm diameter to prepare RSP-1 and RSP-2 specimens. However, a separate mandrel of the same diameter with sides machined is used for the RSP-3-1 and RSP-3-2 design to obtain the flat area in the gauge section. Because in the RSP-3 design, the gauge area is flat instead of circular, as shown in Figure 4c,d. For the RSP3-2 design, to obtain the uniform thickness distribution, the flat gauge section external compression is applied, followed by the wrapping of the shrink tap as shown in Figure 10b,c.

Hoop winding is carried out at an angle of α = 89.60° (Figure 10a), with a fiber-winding rate of 400 mm/s. Five layers are winded, producing a specimen thickness of about 1.6 mm. A tension of 3 kg is also applied to the winding rovings to consolidate the resin better and obtain a higher volume fraction, which results in higher mechanical properties. Fiber tension plays a vital role in the filament winding process. The increase in fiber tension during the filament winding helps in better squeezing out of the resin, which results in a more compact cross-section. Higher compaction increases the fiber volume fraction, ensuing in higher burst pressure and gravimetric efficiency [27]. The fiber volume fraction achieved is approximately 61% and is determined with fiber weight fraction using Equation (2) [28]

(2) $$V_{f}=\frac{1}{\left(1+\frac{\rho_{f}}{\rho_{r}}\left(\frac{1}{w_{f}}-1\right)\right)}$$

where, $w_{f}$ = fiber weight fraction, $\rho_{f}$ = density of fiber and $\rho_{r}$ = density of resin.

The specimens are cured after the wet filament winding. The curing of Elium^®^ 591/carbon specimens is conducted in two stages. First is the UV curing during the winding process, and second is thermal curing at 85 °C for two hours. The Epoxy/carbon specimens are only thermally cured for six hours, as shown in Figure 10d. After curing, specimens are cut using a diamond cutter. The notches are machined for RSP-1 design specimens using the surface grinder.

**Figure 11:**
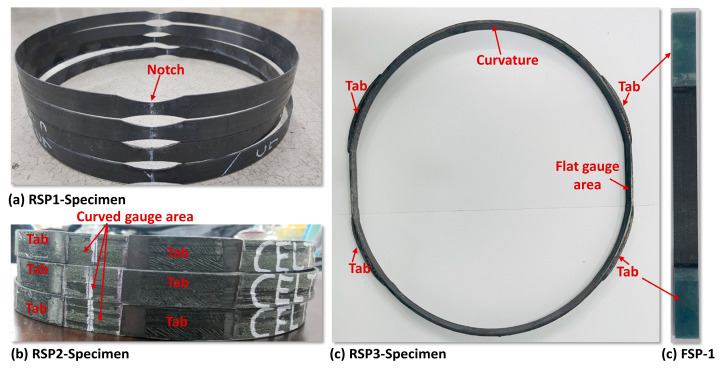
Configuration of manufactured Specimen.

The tabs for RSP-2 and RSP-3 specimens are manufactured with the same material to avoid any possible stress concentration due to the dissimilarity of the material’s stiffness. The rings for the tabs are also manufactured with a filament winding process. The tabs have the same cross-sectional area as the specimen, and their length is 50 mm. At both ends of the tabs, a taper of 10° is also machined to prevent stress concentration and to ensure smooth load transfer to the gauge section. The tabs are then bonded and cured according to the resin type. The surface of the gauge section area is also refined using fine emery paper to install strain gauges. The configurations of the manufactured specimens are shown in Figure 11.

A flat plate specimen (FSP-1) is also manufactured with the filament winding process using the rectangular flat plate mandrel as shown in Figure 3a. The rest of the winding process is the same as depicted in Figure 9 and Figure 10.

## 4. Experiment

### 4.1. The Split Disk Testing

A universal testing machine of 100 KN capacity is used to characterize the ring tensile specimen. The tests are conducted according to the ASTM D2290 [1] standard. This method loads ring specimens through a self-aligning disk test fixture. The test fixture is designed to minimize the effect of the bending moment. This bending moment results from a change in the contour of the ring between two disk sections as they separate.

**Figure 12:**
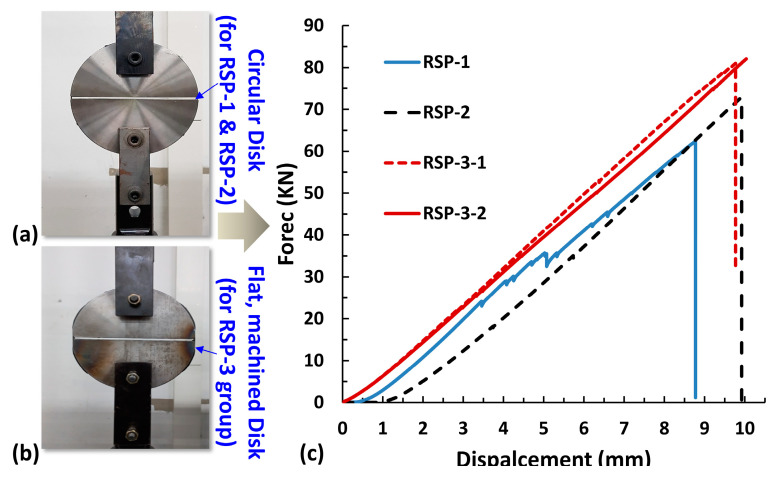
(**a**) Split disk for RSP-1 and 2. (**b**) Split disk for RSP-3. (**c**) Load Displacement curves.

Different split disks are used for different types of designs of specimens, such as for RSP-1 and RSp-2 testing disks are completely round, as shown in Figure 12a. For the RSP-3-design group (RSP-3-1 and RSP-3-2), the testing disks with flat machined gauge sections are used, as indicated in Figure 12b. The utmost care has been taken to ensure that the specimens were loaded in the vertical direction. We have developed a self-aligned split disk design where the disks align themselves during vertical loading. Prior to the actual test, we ensured that the specimen was positioned at the midpoint of the upper and lower disks. Additionally, a vertical pretension force was applied, causing the upper and lower disks, as well as the specimen, to align themselves. Specimens were loaded until failure with a 3 mm/minute loading rate, as specified by the standard. At least three specimens of each design and each material group are tested, and their failure load and apparent hoop tensile strengths are determined and listed in Table 2. The apparent hoop tensile strength of the specimen was determined using Equation (3)

(3) $$\sigma_{a}=\frac{P_{b}}{{2A}_{m}}$$

where *σ_a_* is the ultimate hoop tensile strength, $P_{b}$ is the maximum breaking load, $A_{m}(2*w*t)$ is the minimum cross-sectional area of the two gauge section measurements.

### 4.2. Testing Results

This research aims for two purposes: first, to determine the pros and cons of different designs for the ring tensile specimen, and second, to characterize the carbon/Elium^®^ 591, a thermoplastic composite manufactured with the filament winding process. Three design groups of ring tensile specimens are considered for testing and evaluation, such as RSP-1, RSP-2, and RSP-3 (RSP-3-1, RSP3-2); detailed configurations are illustrated in Figure 4. To evaluate each design group, ring tensile specimens are manufactured with carbon/Elium^®^ 591 and carbon/Epoxy composite using a filament winding process, which also provides a comprehensive comparison of the properties of the thermoplastic and thermosetting composite. In addition to the ring specimen, flat specimens (FSP-1) are also manufactured and tested for comparison purposes. The failure load, tensile strength, and specimen specifications of all tested specimens are listed in Table 2.

Load-displacement curves of three-ring design groups are shown in Figure 12c. The curves of the RSP-1 and RSP-2 specimens are nonlinear at the start, and for the RSP-3 design group (RSP-3-1 and RSP3-2), it is almost linear. Because the gauge section in the first two designs is circular and due to curvature, a bending moment is generated at the gauge section, which causes the nonlinearity on the load-displacement curve in the starts. On the other hand, the gauge section of the RSP-3 design group is flat, and there is not much significant bending moment.

**Figure 13:**
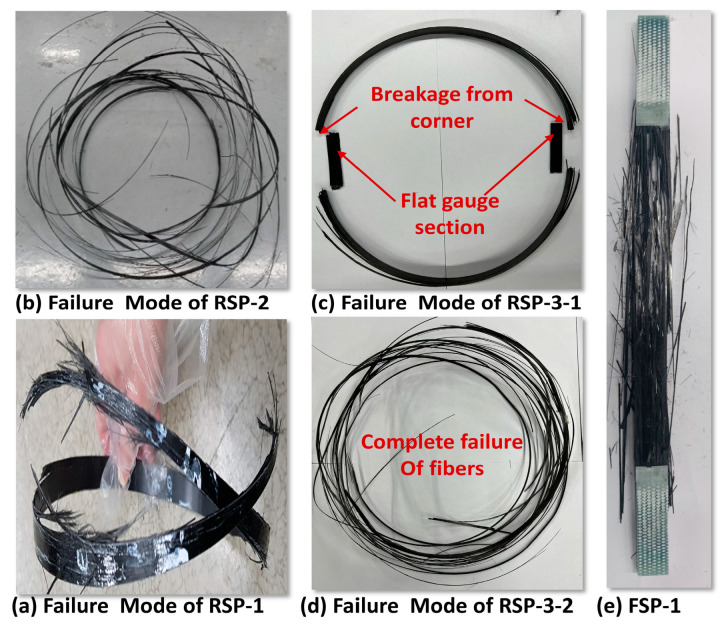
(**a**) Failure mode of RSP-1 specimen (**b**) Failure mode of RSP-2 specimen (**c**) Failure mode of RSP-3-1 specimen (**d**) Failure mode of RSP-3-2 specimen (**e**) Failure mode of FSP-1 specimen.

Failure modes of all the tested specimens are shown in Figure 13. In the RSP-1 design, the specimen did not wholly fail from the gauge section. Instead, the specimen is split starting from the notch radius and propagating towards the hoop direction. This propagation is due to shear strain between fibers that causes the specimen to fail without complete breakage of the fibers due to tensile load at the gauge section, which results in a lower load-carrying capacity. As a result of the shear failure of the matrix between the fibers, the load-displacement curve for the RSP-1 design (notched specimen) also reveals many wrinkles, as shown in Figure 12c. On the other hand, the load-displacement curves of the RSP-2 and RSP-3 designs are wrinkle-free, and the complete failure of the fibers at the gauge section under the tensile load was observed. Such a failure mode is necessary to ensure a higher tensile load-carrying capacity.

However, the failure mode of RSP-3-1 is different from RSP3-2, as shown in Figure 13c,d, because, in RSP-3-1, the corner thickness (transition region) is less than the remaining gauge section, which results in low bending stiffness. Furthermore, the sharp corner is also prone to stress concentrations. Due to these two effects, the flat gauge section gets separated from the specimen during the testing, as shown in Figure 13c, resulting in low load-carrying capacity. In contrast, the failure of specimen RSP-3-2 is different. All fiber failed in the hoop direction, which produces the higher strength. Similarly, in the failure mode for the flat plate (FSP-1) specimen, all fiber failed in the gauge section in the loading direction as shown in Figure 13e.

**Figure 14:**
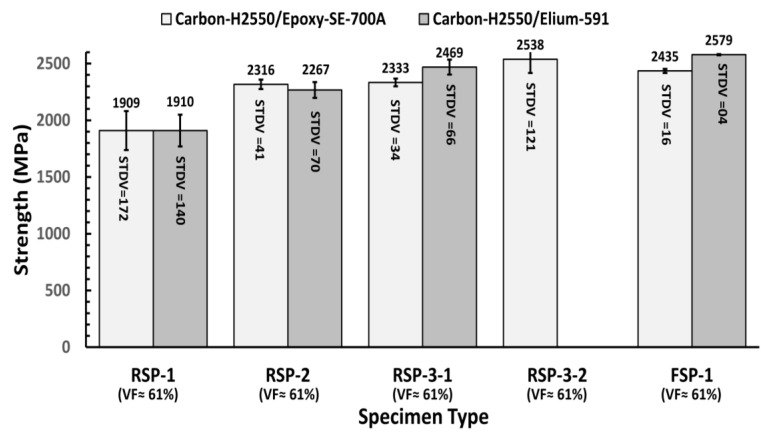
Hoop tensile strength comparison of Carbon-H2550/Elium^®^ 591 and Carbon-H2550/epoxy-SE-700A specimen.

The average hoop tensile strengths for each specimen design and material group are presented in Figure 14. To enable meaningful comparison with other ring specimens, the tensile strength results of the flat plate specimen (FSP-1) have been adjusted to a 61% volume fraction, despite its actual volume fraction being 51%. The hoop tensile strength values of each tested specimen are also listed in Table 2.

The experimental results demonstrate that the RSP-1 design, featuring a notch at the gauge section, exhibits a lower predicted strength compared to the RSP-2 and RSP-3 designs. This disparity can be attributed to the presence of an 85-mm radius notch in the gauge section area of the specimen. The numerical study already revealed that this notch introduces stress concentration, resulting in in-plane shear strain and premature splitting of the specimen in the hoop direction before the fibers reach the point of breakage. Specifically, the average strengths for carbon/epoxy and carbon/Elium^®^ 591 specimens are reported as 1909 MPa and 1910 MPa, respectively. These values are accompanied by corresponding standard deviations of 172 MPa and 140 MPa.

The average strength values of RSP-2 specimens for carbon/epoxy and carbon/Elium^®^ 591 specimens are 2316 and 2267, with a standard deviation of 41 and 70 MPa, respectively. It indicates that the RSP-2 design demonstrated higher strength with more consistency than the RSP-1. The RSP-2 specimen has a uniform stress distribution in the gauge area along the width direction compared to the RSP-1 (notched specimen), as confirmed by the numerical study’s results illustrated in Figure 7b. However, the experimental tensile strength of the RSP-2 specimen is less than the RSP-3 specimen (designed with a flat gauge section), as demonstrated in Figure 14. Although the RSP-2 design has a uniform stress distribution along the width direction, stress distribution along the thickness direction is still not uniform. The RSP-3 design group is the only one to ensure uniform stress distribution in all directions throughout the gauge section, as shown in Figure 7c.

The split disk test results from Figure 14 show that the RSP-3 specimens demonstrated higher load-carrying capacity than all other designs. The average tensile strength of the RSP-3-1 specimen made of carbon-H2550/Elium^®^ 591 is 2469 MPa, which is 10% more than the RSP-2 specimen of the same material. However, the strength of the epoxy specimen is almost the same as the RSP-2 design. Because in the RSP-3-1 design, there are sharp corners in the transition region, and due to stress concentration flat gauge area is separated from the specimen during the testing, as shown in Figure 13c, which results in lower load-carrying capacity. However, this ring design deficiency is rectified in the RSP3-2 ring design, and a smooth transition between the flat gauge section and curved specimen region is provided, which produces a higher strength. as shown in Figure 14. Epoxy-carbon specimens are tested with RSP-3-2 specimen, and the average strength is 2538 Mpa which is 10.9% higher than Epoxy-carbon specimens of RSP-2 ring design.

**Figure 15:**
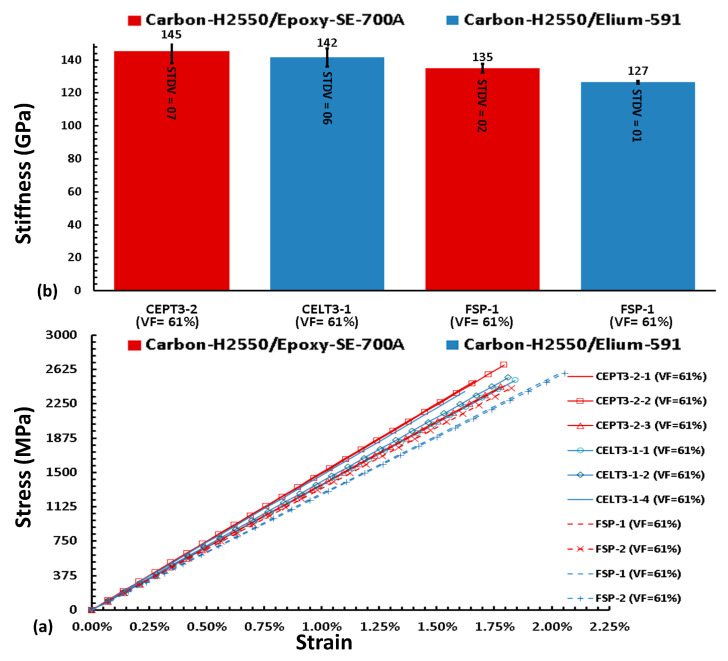
(**a**) Elastic Modulus values with standard deviation (**b**) Stress-strain curve of Carbon-Epoxy and Carbon-Elium specimens.

The stress-strain curves and the stiffens data are also plotted in Figure 15. The average stiffness is 145 GPa and 141 Gpa for Carbon/Epoxy and Carbon/Elium specimens, respectively, are measured using ring tensile specimen RSP-3 (with 61% VF) specimen design. Flat specimens (FSp-1) are also tested; their average stiffness is 135 GPa and 127 Gpa (with VF = 61%). However, the lower volume fraction is a serious matter of concern in using the flat specimen. This experimental study also validates that the mechanical properties of Elium^®^ 591-based composite are equivalent to epoxy-composite, and due to its additional attributes, such as recyclability and short curing time, it can be used to manufacture hydrogen tanks.

## 5. Conclusions

Carbon neutrality has made hydrogen tanks increasingly popular in recent years. An efficient design of high-performance tanks depends upon the accuracy of input material attributes. However, as measured by the conventional notched specimen, the material properties are always lower than the actual Values. Current research proposes alternate designs of ring tensile specimens for accurate and reliable characterization of filament wound structure. The alternative designs’ pros and cons are also discussed using numerical and experimental studies. Additionally, the characterization of the Elium^®^ 591 resin-based composite was also performed. It is a liquid thermoplastic resin, and a tank made with it can be recycled at the end of life. Based on the current research, the following conclusions have been drawn.

- The RSP-1 design represents the conventional notched design of a ring tensile specimen. However, the stress distribution within the notched specimen is non-uniform, with a stress concentration occurring in the notched region. This stress concentration leads to in-plane shear stress, causing the specimen to split in the hoop direction much earlier than fiber breakage. As a result, the predicted tensile strength is lower than the actual value.
- The RSP-2 design eliminates the notch and instead incorporates tabs to achieve a uniform stress distribution in the gauge area. Numerical analyses of this design show that there is a uniform stress distribution in the gauge section along the width direction. However, there is some non-uniformity along the thickness direction due to local bending. Despite this, the RSP-2 design is relatively easy to manufacture, and experimental results consistently demonstrate performance approximately 21% better than the traditional notched specimen.
- The RSP-3 design group, consisting of RSP-3-1 and RSP-3-2, eliminates the notch and incorporates a flat gauge section. Tabs are also introduced to prevent stress concentration and direct failure towards the gauge section. Numerical simulations demonstrate that only RSP-3 exhibits a uniform hoop stress distribution throughout the gauge section in all directions, which is crucial for achieving a higher load-bearing capacity. Experimental results support these findings, indicating that the proposed specimen can carry a 30% higher load compared to the conventional notched specimen. However, it is worth noting that this design presents challenges in terms of manufacturing due to the flat gauge section, and the non-uniform thickness distribution at the gauge section needs to be addressed.
- The FSP-1 (Flat plate specimen) has a lower volume fraction compared to the ring specimen. To increase the volume fraction, autoclave pressurizing is required.
- The Carbon-H2550/Elium^®^ 591 composite, produced through filament winding, undergoes characterization and is compared with the conventional Carbon/Epoxy composite. The findings reveal that the strength of the Elium^®^ 591-based composite is comparable to that of the traditional epoxy composite. Moreover, additional attributes such as recyclability make the Elium^®^ 591-based composite a compelling option for hydrogen tank manufacturing.

## Figures and Tables

**Table 1:** Pros and Cons of different types of tensile specimens.

Type	Merits	Demerits
RSP-1	Good for isometric material and biaxially reinforced composite material Use a notch to concentrate the failure at the gauge section Winding on the circular mandrel Volume fraction ≥ 60%	Stress concentration around the notch Not suitable for Unidirectional (UD) reinforced composite Localized bending at the gauge section Predict 20 to 30% lower tensile strength
RSP-2	Strength is greater than RSP-1. Use tabs instead of a notch to concentrate the failure at the gauge section. Suitable for UD-reinforced composite to determine the tensile strength and tensile modulus Easy manufacturing (Filament winding on the circular mandrel) Volume fraction ≥ 60%	Non-uniform stress distribution along the width direction due to localized bending imposed at the gauge section due to curvature. Results in about 10% lower prediction of the tensile strength
RSP-3-1	uniform stress distribution in all directions Use tabs instead of a notch to concentrate the failure at the gauge section. Localized bending is controlled by providing the flat gauge section Suitable for UD-reinforced composite to determine the tensile strength and tensile modulus Filament winding on the circular stepped mandrel Volume fraction ≥ 60%	The abrupt transition between the circular region and the flat gauge section produces the stress concentration, leading to a lower tensile strength prediction. Non-uniform thickness variation in the gauge section
RSP-3-2	No stress concentration as there as the transition between the flat gauge section and the circular region is smooth (a radius of 50 mm is provided) Use tabs instead of a notch to concentrate the failure at the gauge section Uniform stress distribution in all directions Localized bending is controlled by providing the flat gauge section Suitable for UD-reinforced composite to determine the tensile strength and tensile modulus Filament winding on the circular stepped mandrel Volume fraction ≥ 60% Produces higher strength	Non-uniform thickness variation in the gauge section Non-uniform thickness can be controlled by applying the external compressive force at the flat gauge region during the curing process
FSP-1	Filament winding on the plate mandrel No stress concentration or localized bending Easy to manufacture	Low volume fraction ≈ 50% The autoclave is required to increase the volume fraction

**Table 2:** Specification of all tested specimens.

Type	Material	Specimen ID	W (mm)	T (mm)	Area	Failure Load (KN)	Tensile Strength (MPa)	VF (%)
RSP-1	H2550/Epoxy	CEPT1-1	9.50	1.60	30.40	64.07	2108	61
		CEPT1-2	9.50	1.60	30.40	55.02	1810	
		CEPT1-3	9.50	1.60	30.40	55.06	1811	
	H2550/Elium 591	CELT1-1	9.70	1.60	31.04	62.40	2010	61
		CELT1-2	9.70	1.60	31.04	54.32	1750	
		CELT1-3	9.70	1.60	31.04	61.10	1968	
RSP-2	H2550/Epoxy	CEPT2-1	10.80	1.60	34.56	81.56	2360	61
		CEPT2-2	10.10	1.60	32.32	74.70	2311	
		CEPT2-3	10.00	1.60	32.00	72.89	2278	
	H2550/Elium 591	CELT2-1	10.10	1.60	32.32	70.95	2195	61
		CELT2-2	10.15	1.60	32.48	73.74	2270	
		CELT2-3	10.85	1.60	34.72	81.07	2335	
RSP-3-1	H2550/Epoxy	CEPT3-1-1	9.83	1.60	31.46	72.47	2304	61
		CEPT3-1-2	10.45	1.60	33.44	77.21	2309	
		CEPT3-1-3	9.66	1.60	30.91	72.40	2342	
		CEPT3-1-4	9.73	1.60	31.14	74.00	2377	
	H2550/Elium 591	CELT3-1-1	10.11	1.60	32.35	81.00	2504	61
		CELT3-1-2	10.12	1.60	32.38	82.08	2535	
		CELT3-1-3	10.00	1.60	32.00	76.35	2386	
		CELT3-1-4	9.53	1.60	30.50	74.74	2451	
RSP-3-2	H2550/Epoxy	CEPT3-2-1	10.58	1.60	33.86	84.88	2507	61
		CEPT3-2-2	11.25	1.60	36.00	96.20	2672	
		CEPT3-2-3	10.64	1.60	34.05	82.94	2436	
FSP-1	H2550/Epoxy	CEPT4-1	15.00	1.30	19.50	39.91	2047	51
		CEPT4-2	15.00	1.30	19.50	39.49	2025	
	H2550/Elium 591	CELT4-1	15.00	1.30	19.50	41.98	2153	51
		CELT4-2	15.00	1.30	19.50	42.10	2159	

## Data Availability

Not applicable.

## References

[1] (2016). Standard Test Method for Apparent Hoop Tensile Strength of Plastic or Reinforced.

[2] Kaynak C., Erdiller E.S., Parnas L., Senel F. (2005). Use of split-disk tests for the process parameters of filament wound epoxy composite tubes. Polym. Test..

[3] Zhao Y., Druzhinin P., Ivens J., Vandepitte D., Lomov S.V. (2021). Split-disk test with 3D Digital Image Correlation strain measurement for filament wound composites. Compos. Struct..

[4] Lee S., Kim S.-H., Kim S., Choi J., Choi H.-J. (2018). Hoop tensile strength of tubular carbon fiber reinforced silicon carbide matrix composites. Ceram. Int..

[5] Abed G.M.H., Pinna C., Foreman J.P., Hayes S.A., Correlation D.I. Characterisation of the Tensile and Fracture Properties of Filament Wound Carbon Fibre Rings. Proceedings of the ECCM16—16th European Conference on Composite Materials.

[6] Ramesh G., Gettu R., Bharatkumar B.H. (2016). Modified split disk test for characterization of FRP composites. J. Struct. Eng..

[7] Charan V.S., Vardhan A.V., Raj S., Rao G.R., Rao G.V., Hussaini S.M. (2019). Experimental characterization of CFRP by NOL ring test. Mater. Today Proc..

[8] Mertiny P., Ellyin F. (2002). Influence of the filament winding tension on physical and mechanical properties of reinforced composites. Compos. Part A Appl. Sci. Manuf..

[9] Naseva S., Srebrenkoska V., Risteska S., Stefanovska M., Srebrenkoska S. (2015). Mechanical Properties of Filament Wound Pipes: Effects of Winding Angles. Qual. Life.

[10] Laiarinandrasana L., Devilliers C., Oberti S., Gaudichet E., Fayolle B., Lucatelli J.M. (2011). Ring tests on high density polyethylene: Full investigation assisted by finite element modeling. Int. J. Press. Vessel. Pip..

[11] Hwang T.-K., Park J.-B., Kim H.-G. (2012). Evaluation of fiber material properties in filament-wound composite pressure vessels. Compos. Part A Appl. Sci. Manuf..

[12] Kim Y., Choi C., Kim C.-G., Doh Y.-D. (2016). Ring burst test of filament wound composites for environmental resistance. J. Compos. Mater..

[13] Bois C., Pilato A., Wahl J.-C., Perry N. (2013). Proposal for a smart pressurised ring test to study thick composite produced by filament winding. Compos. Part B Eng..

[14] Pinto T.H.L., Gul W., Torres L.A.G., Cimini C.A., Ha S.K. (2021). Experimental and numerical comparison of impact behavior between thermoplastic and thermoset composite for wind turbine blades. Materials.

[15] https://www.arkema.com/global/en/products/product-finder/product/incubator/elium/elium-resin-for-composite-hydrogen-tanks/%0A.

[16] (2017). Standard Test Method for Tensile Properties of Polymer Matrix Composite Materials.

[17] Roh H.S., Hua T.Q., Ahluwalia R.K. (2013). Optimization of carbon fiber usage in Type 4 hydrogen storage tanks for fuel cell automobiles. Int. J. Hydrogen Energy.

[18] Alcántar V., Aceves S.M., Ledesma E., Ledesma S., Aguilera E. (2017). Optimization of Type 4 composite pressure vessels using genetic algorithms and simulated annealing. Int. J. Hydrogen Energy.

[19] Hua T.Q., Roh H.-S., Ahluwalia R.K. (2017). Performance assessment of 700-bar compressed hydrogen storage for light duty fuel cell vehicles. Int. J. Hydrogen Energy.

[20] Mahdavi H., Rahimi G.H., Farrokhabadi A. (2018). Failure Analysis of (±55°)_9_ Filament-Wound GRE Pipes Using Explicit Finite Element Method: A Comparison with the Experimental Method. J. Fail. Anal. Prev..

[21] Tsukizoe T., Ohmae N. (1983). Friction and wear of advanced composite materials. Fibre Sci. Technol..

[22] Wan Y.Z., Chen G.C., Raman S., Xin J.Y., Li Q.Y., Huang Y., Wang Y.L., Luo H.L. (2006). Friction and wear behavior of three-dimensional braided carbon fiber/epoxy composites under dry sliding conditions. Wear.

[23] Ruggiero A., Merola M., Carlone P., Archodoulaki V.-M. (2015). Tribo-mechanical characterization of reinforced epoxy resin under dry and lubricated contact conditions. Compos. Part B Eng..

[24] Alves M.P., Gul W., Cimini Junior C.A., Ha S.K. (2022). A Review on Industrial Perspectives and Challenges on Material, Manufacturing, Design and Development of Compressed Hydrogen Storage Tanks for the Transportation Sector. Energies.

[25] Arkema https://www.arkema.com/france/en/.

[26] Hyosung Advanced Materials Corporation http://www.hyosungadvancedmaterials.com/resources/front/kr/files/tansome_catalog_2020.pdf.

[27] Błachut A., Wollmann T., Panek M., Vater M., Kaleta J., Detyna J., Hoschützky S., Gude M. (2023). Influence of fiber tension during filament winding on the mechanical properties of composite pressure vessels. Compos. Struct..

[28] Bhudolia S.K., Perrotey P., Joshi S.C. (2017). Optimizing polymer infusion process for thin ply textile composites with novel matrix system. Materials.

